# Tribological Behavior and Self-Lubrication Mechanisms of C*_f_*/SiC-B_12_(C,Si,B)_3_ Composites Under Coupled Temperature-Velocity Conditions: A Preliminary Study

**DOI:** 10.3390/ma19091703

**Published:** 2026-04-23

**Authors:** Xiaoyang Guo, Shuaixu Chun, Haifeng Nie, Xuxin Ping, Jingchen Yuan, Quanxing Ren, Yan Jiang, Zhengren Huang, Qing Huang, Yinsheng Li

**Affiliations:** 1 School of Materials Science and Engineering, Shenyang University of Chemical Technology, Shenyang 110142, China; guoxiaoyang@nimte.ac.cn; 2 Zhejiang Key Laboratory of Data-Driven High-Safety Energy Materials and Applications, Ningbo Key Laboratory of Special Energy Materials and Chemistry, Ningbo Institute of Materials Technology and Engineering, Chinese Academy of Sciences, Ningbo 315201, China; chunshuaixu@nimte.ac.cn (S.C.); nie_haifeng@126.com (H.N.); pxx17863523904@163.com (X.P.); yuanjingchen@nimte.ac.cn (J.Y.); zhrhuang@nimte.ac.cn (Z.H.); huangqing@nimte.ac.cn (Q.H.); 3 Qianwan Institute of CNITECH, Ningbo 315336, China; 4 China Testing & Certification International Group Shandong Co., Ltd., Zaozhuang 277100, China

**Keywords:** C*_f_*/SiC-B_12_(C,Si,B)_3_ composite, microstructure, friction, wear resistance, self-lubrication

## Abstract

To address the increasing demands for lightweight, high-temperature resistant braking materials under extreme service conditions, a novel C*_f_*/SiC-B_12_(C,Si,B)_3_ composite was developed in this work. The composite was fabricated via a hybrid slurry infiltration-reactive melt infiltration (SI-RMI) process. The tribological performance under coupled temperature–velocity conditions was systematically evaluated using a ball-on-disk tester over temperatures from 25 to 600 °C (at 900 r/min) and sliding speeds from 300 to 900 r/min (at 600 °C). The results indicate that temperature dominates the friction and wear behavior. At room temperature, the composite exhibits a friction coefficient of 0.52 and a wear rate of 4.019 × 10^−4^ mm^3^/(N·m). With increasing temperature, friction coefficients decreased to 0.43 at 400 °C and 0.41 at 600 °C, while wear rates increased sharply to 12.025 × 10^−4^ mm^3^/(N·m) at 400 °C before declining to 5.228 × 10^−4^ mm^3^/(N·m) at 600 °C. Under the fixed temperature of 600 °C, raising rotational speed from 300 to 900 r/min increased the wear rate only marginally (4.953 to 5.228 × 10^−4^ mm^3^/(N·m)). Surface analysis indicates that a continuous Si-B-O oxide layer (mainly SiO_2_ and B_2_O_3_) forms at 600 °C, which may provide solid lubrication and oxidation resistance. The present work offers a preliminary exploration of the tribological evolution and self-lubrication mechanisms of C*_f_*/SiC-B_12_(C,Si,B)_3_ composites, providing potential insights for the design of advanced ceramic-matrix braking materials.

## 1. Introduction

Braking safety is a fundamental and critical requirement in modern ground transportation and aerospace, receiving stringent regulatory oversight and extensive research attention. The demand for high performance braking systems has intensified in applications such as high-speed rail, heavy duty vehicles, aircraft, and next-generation electric vehicles, driven by increasingly strict safety standards and operational requirements especially the requirement for shorter braking distances under extreme service conditions [1,2]. A stable friction coefficient is critical for reliable braking performance, with high-end brake materials typically requiring the coefficient to remain between 0.3 and 0.6 [3,4,5,6]. During emergency braking, massive kinetic energy is instantly converted into heat, often driving the temperature at friction interfaces above 400 °C [7]. Conventional metallic brake materials suffer severe thermal fade at elevated temperatures, with the friction coefficient plummeting below 0.2 and wear rates surging sharply [8]. Additionally, their inherent drawbacks-including high density (>7 g/cm^3^, large thermal expansion coefficient, and poor oxidation resistance-significantly limit their application under high-energy and long-service-life operating conditions.

Carbon fiber-reinforced silicon carbide (C*_f_*/SiC) composites have emerged as highly promising candidate materials owing to their low density (<2.8 g/cm^3^), moderate oxidation resistance, as well as excellent high-temperature stability and wear resistance superior to those of metals [9,10,11,12,13,14,15,16]. These advantages make them ideal for critical braking applications such as aircraft brake disks, high-speed-train brake pads, and high-performance automotive braking systems [17,18,19]. However, during emergency braking, the rapid temperature rise can lead to accelerated oxidation of carbon fibers and agglomerated residual silicon within the composite. This phenomenon results in significant degradation of mechanical properties and a dramatic increase in wear rate, limiting their broader application [7,20,21].

To further improve the tribological properties and simultaneously achieve the two core indicators of braking safety moderate friction coefficient and low wear rate the introduction of second-phase particles with high hardness, excellent high-temperature stability and potential self-lubricating properties into C*_f_*/SiC composites has become a research hotspot [22]. Among various candidate materials, boron carbide (B_4_C) has attracted extensive attention. Its unique B_12_ icosahedral structure and strong covalent bonding network endow it with low density and exceptional hardness, and it can oxidize in situ to form lubricious B_2_O_3_ at elevated temperatures [23,24,25,26,27,28]. For instance, Fan et al. [22] and Li et al. [29] reported that B_4_C oxidizes to form fluid B_2_O_3_ during high-speed sliding, which acts as a solid lubricant and simultaneously inhibits oxygen inward diffusion, thereby reducing wear. Zhang et al. [30,31,32,33] observed that the hardness mismatch between B_4_C and SiC leads to preferential wear of SiC, causing B_4_C to protrude and form an in situ textured surface that reduces the real contact area and traps wear debris, further lowering wear. However, under severe thermo-mechanical coupling, the B_4_C phase is prone to excessive oxidation, spalling, brittle fracture, and weak interfacial bonding with the SiC matrix, which compromises the integrity and durability of the friction film [34,35,36,37,38].

Recent studies have shown that during reactive melt infiltration with silicon, B_4_C can react with molten Si via a dissolution-precipitation mechanism to form a ternary B-C-Si phase, denoted as B_12_(C,Si,B)_3_ [39,40]. Crystallographically, this phase exhibits an icosahedral structure similar to that of B_4_C, accommodating Si, B, and C atoms in its lattice. It has been reported that B_12_(C,Si,B)_3_ retains high hardness while mitigating the intrinsic brittleness of B_4_C [41,42,43]. Upon oxidation at elevated temperatures, it forms a Si-B-O oxide layer composed of SiO_2_ and B_2_O_3_. Such an oxide film is expected to provide excellent solid lubrication and oxidation resistance, enabling a favorable synergy between oxidation resistance and tunable tribochemical behavior. It is therefore hypothesized that the incorporation of B_12_(C,Si,B)_3_ into C*_f_*/SiC composites can alleviate the common performance trade-off observed in conventional B_4_C-modified systems, allowing the material to achieve a moderate friction coefficient and a low wear rate simultaneously over a wide temperature range. Nevertheless, the tribological evolution of the B_12_(C,Si,B)_3_ phase under coupled temperature–velocity conditions remains poorly understood and warrants systematic investigation.

In this study, a novel C*_f_*/SiC-B_12_(C,Si,B)_3_ composite was fabricated via a hybrid process of slurry infiltration combined with reactive melt infiltration (SI-RMI). Using a ball-on-disk tribometer, the tribological behavior of the composite was systematically characterized under variable temperature conditions (25, 400, 600 °C) and various sliding speeds (300, 600, 900 r/min). The intrinsic lubrication and wear mechanisms were elucidated by analyzing the evolution of the friction coefficient, wear rate, surface chemical composition, and microstructure. The novelty of this work lies in the systematic investigation into the tribological properties of this unique composite under coupled temperature–velocity conditions, which fills a critical gap in the existing literature. This study aims to provide a theoretical basis for the design of high-performance customizable ceramic matrix braking materials for extreme service conditions.

## 2. Experimental Procedure

### 2.1. Raw Materials

The following raw materials were employed in this study. Carbon fiber preform (2.5D, T300, Density: 0.45 g/cm^3^, Porosity: 75%, Tianniao High Technology Co., Ltd., Wuxi, China); B_4_C powders with two different particle sizes (D_50_ = 0.6 μm and D_50_ = 8.6 μm, purity: 95.00%, China Boron Technology Co., Ltd., Weihai, China); Carbon black (D_50_ = 100 nm, Purity: 99.5%, Qinhuangdao Yinuo High-Tech Material Development Co., Ltd., Qinhuangdao, China); Tetramethylammonium hydroxide (TMAH, Purity: 25% aqueous solution, Sinopharm Chemical Reagent Co., Ltd., Shanghai, China); Polyvinyl pyrrolidone (PVP, Purity: 99.5%, Sinopharm Chemical Reagent Co., Ltd., Shanghai, China); Silicon particles (D_50_ = 1 mm, Purity: 99.99%, Qinhuangdao Yinuo High-tech Material Development Co., Ltd., Qinhuangdao, China).

### 2.2. Composite Fabrication

The carbon fiber preform was first desized by heating at 900 °C for 1 h in vacuum, followed by graphitization at 1900 °C for 2 h in argon. It was then transferred into a chemical vapor infiltration (CVI) furnace, where a pyrolytic carbon (PyC) interphase (~0.5 μm) and a SiC interphase (~1.5 μm) were sequentially deposited onto the fiber surfaces to form a hybrid PyC/SiC interphase. The resulting preform exhibited an open porosity of approximately 55%.

A slurry was prepared by dispersing B_4_C powders (volume ratio of 8.6 μm:0.6 μm = 7:3) and carbon black (volume ratio of B_4_C to carbon black = 9:1) in deionized water. Tetramethylammonium hydroxide (TMAH) and polyvinyl pyrrolidone (PVP) were added as dispersants for B_4_C and carbon black, respectively. The solid content of the slurry was adjusted to 40 vol%, followed by ball milling at 200 r/min for 12 h using SiC milling media (5 mm in diameter) with a ball-to-powder mass ratio of 3:1 to achieve uniform dispersion.

The preform with the PyC/SiC interphase was immersed in the slurry and infiltrated under a nitrogen pressure of 2 MPa for 30 min. After infiltration, the sample was dried at 80 °C for 12 h and then pyrolyzed at 800 °C under vacuum for 1 h to remove organic components, yielding a porous C*_f_*/C-B_4_C preform.

**Figure 1:**
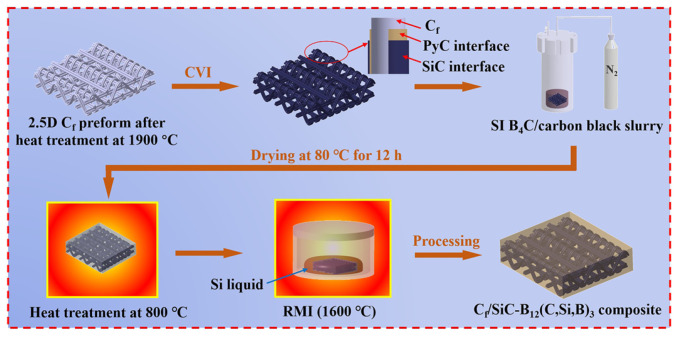
Schematic illustration of the preparation process for C*_f_*/SiC-B_12_(C,Si,B)_3_ composites.

The preform was embedded in silicon powder inside a graphite crucible and subjected to reactive melt infiltration (RMI) densification at 1600 °C for 1 h under vacuum. After densification, residual silicon on the surface was removed by grinding, followed by polishing with 2000-grit sandpaper, yielding the C*_f_*/SiC-B_12_(C,Si,B)_3_ composite. A schematic diagram of its preparation process is presented in Figure 1. The initial surface roughness of the C*_f_*/SiC-B_12_(C,Si,B)_3_ composite specimens before friction testing was measured using a laser confocal microscope (VK-X1000, Keyence, Osaka, Japan). The average arithmetic mean roughness (R_a_) was determined to be 0.63 ± 0.09 μm based on five independent measurements.

### 2.3. Density and Porosity Measurement

The bulk density and open porosity of the composite were determined using the Archimedes displacement method with deionized water as the immersion medium.

### 2.4. Residual Silicon Content Measurement

The residual silicon content in the sintered C*_f_*/SiC-B_12_(C,Si,B)_3_ composite was determined by a mixed-acid etching method [44]. The sample was immersed in a mixed acid solution (HNO_3_:HF = 3:2 by mass) for 24 h to selectively dissolve residual silicon. The volume fraction of residual silicon was calculated using the following equation:

(1) $$V_{Si}=\frac{\left(m_{1}\cdot m_{2}\right)\cdot \rho }{2.33\cdot m_{1}}\times 100\%$$

where $V_{\mathrm{Si}}$ is the volume fraction of residual silicon (vol%), $m_{1}$ and $m_{2}$ are the masses of the sample before and after acid etching (g), respectively, ρ is the density of the composite (g/cm^3^), and 2.33 g/cm^3^ is the density of silicon.

### 2.5. Friction and Wear Testing

Ball-on-disk friction tests were conducted according to GB/T43853-2024 [45] using an MMQ-02G high-temperature tribometer (Jinan Yihua Tribology Testing Technology Co., Ltd., Jinan, China). A SiC ceramic ball (Ø12 mm) was employed as the counterbody against a C*_f_*/SiC-B_12_(C,Si,B)_3_ composite disk specimen with a diameter of 25 mm and a thickness of 5 mm. SiC was selected because it represents the typical counterface material (brake pad) in actual braking applications such as new energy vehicles, where ceramic-based friction pairs are used to achieve lightweight, high-temperature resistance, and superior wear performance. Among the available counterbody options (e.g., GCr15 steel, ZrO_2_, Al_2_O_3_, SiC), SiC most closely mimics the material pairing under real service conditions, ensuring that the measured tribological behavior is relevant to practical applications.

All tests were conducted under a normal load of 100 N for a sliding duration of 600 s at a friction radius of 6.5 mm. Two sets of test conditions were employed:

1. Temperature variation at fixed speed: Tests were carried out at 900 r/min under ambient air at 25 °C (RT, room temperature), 400 °C (MT, medium temperature), and 600 °C (HT, high temperature). The corresponding specimens were labeled RT-900, MT-900, and HT-900.
2. Speed variation at fixed temperature: Tests were conducted at 600 °C with rotational speeds of 300, 600, and 900 r/min, resulting in specimens labeled HT-300, HT-600, and HT-900.

Before each test, the specimen was held at the target temperature for 10 min to ensure thermal equilibrium. The test temperatures of 400 °C and 600 °C were selected based on the typical operating conditions of ceramic-matrix brake materials. During emergency braking, frictional heating can rapidly elevate the brake disk temperature to several hundred degrees Celsius. These temperatures are commonly used as benchmarks in the industry to evaluate the high-temperature tribological performance and thermal stability of braking materials. Thus, investigating the friction and wear behavior at 400 °C and 600 °C is essential to assess the composite’s suitability for practical braking applications.

**Figure 2:**
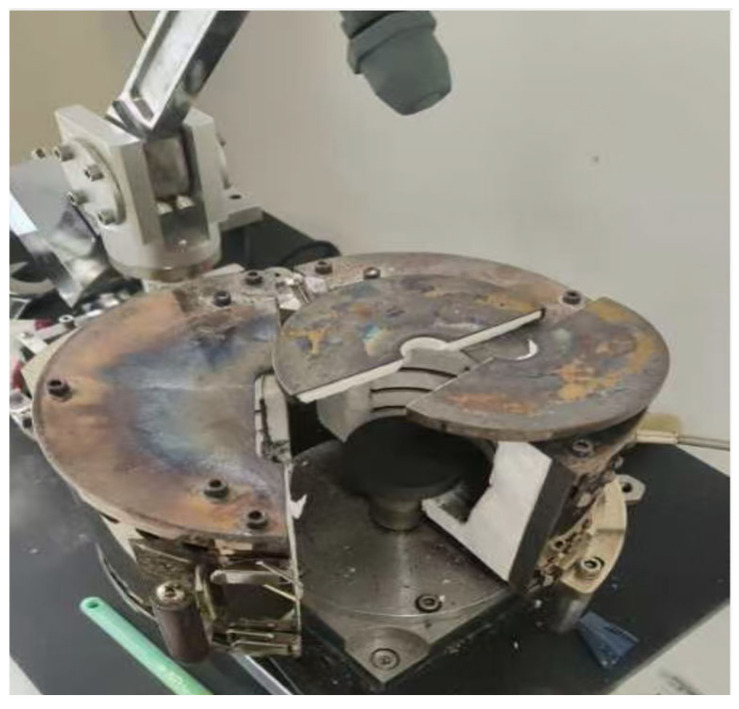
Optical photograph of the high-temperature ball-on-disk tribometer used in this study.

Figure 2 presents a photograph of the high-temperature ball-on-disk tribometer used in this study. The equipment consists of a sealed heating chamber and a rotating disk, providing a relatively isolated testing environment. During the tests, the chamber was closed, and the local high temperatures generated by heating and frictional sliding helped maintain a dry condition.

It should be noted that, due to facility limitations, all friction and wear tests were performed as single measurements under each specified condition. Consequently, this study is intended as a preliminary investigation, and the results should be interpreted with caution regarding statistical variability.

Nevertheless, the ball-on-disk testing method has been shown to yield reproducible results for C*_f_*/SiC composites. For instance, Tang et al. [46] investigated the friction and wear properties of C*_f_*/SiC composites using a similar ball-on-disk configuration with multiple samples. Their reported error bars for both friction coefficient and wear rate were small relative to the mean values, indicating good repeatability of this method. This provides indirect support for the reliability of the present friction and wear data, while the limitation of single measurements is fully acknowledged.

After each friction test, the specimen was immediately sealed in a vacuum bag to prevent hydration of the worn surface. All post-test characterizations, including SEM, XRD, and XPS, were completed within 48 h to preserve the original state of the tribological products on the worn surface.

The volumetric wear rate was calculated from the mass loss using the following equation [47]:

(2) $$W=\;\frac{m_{0}-m_{1}}{L\;\times \;F\;\times \;\rho }$$

where *W* is the volume wear rate of the specimen (mm^3^/(N·m)), *m*_0_ and *m*_1_ are the sample masses before and after testing (g), *ρ* is the composite density (g/cm^3^), *L* is the total sliding distance (m), and *F* is the applied normal load (N).

The total sliding distance L for each test was calculated based on the friction radius (6.5 mm), the rotational speed, and the test duration (600 s). At rotational speeds of 300, 600, and 900 r/min, the total sliding distances are approximately 122.52 m, 245.04 m, and 367.57 m, respectively.

### 2.6. Characterization

X-ray diffractometry (XRD, D8 Advance, Bruker, Karlsruhe, Germany) was used to analyze the phase composition. The test parameters were as follows: voltage, 40 kV; current, 40 mA; 2θ range, 10–90°; time per step, 0.15 s.

Scanning electron microscopy (SEM, SU8230, Hitachi, Tokyo, Japan) combined with energy-dispersive spectroscopy (EDS) was employed to observe the microstructure and elemental distribution. The accelerating voltage and probe current were approximately 20 kV and 10 μA, respectively. For SEM morphology observation, the working distance was approximately 8 mm. For EDS elemental analysis, the working distance was approximately 15 mm, and the EDS acquisition time was 120 s.

X-ray photoelectron spectroscopy (XPS, AXIS SUPRA, Shimadzu, Tokyo, Japan) was used to characterize the chemical states on the worn surfaces.

Laser scanning confocal microscopy (VK-X1000, Keyence, Osaka, Japan) was employed at a magnification of 10× to obtain the surface roughness before friction and the three-dimensional topography of the wear tracks.

Transmission electron microscopy (TEM, Talos F200X, Thermo Fisher Scientific, Waltham, MA, USA) was applied to characterize the crystallographic structure of the B_12_(C,Si,B)_3_ phase.

Raman spectroscopy (LabRAM HR Evolution, Horiba, Kyoto, Japan) was used to analyze the structural changes of carbon phases on the specimen surfaces before and after friction, with a spectral range of 100–2000 cm^−1^.

## 3. Results and Discussion

### 3.1. Phase Composition Analysis

The as fabricated C*_f_*/SiC-B_12_(C,Si,B)_3_ composite exhibited a bulk density of 2.65 g/cm^3^ and an open porosity of 0.46%, indicating a high degree of densification.

**Figure 3:**
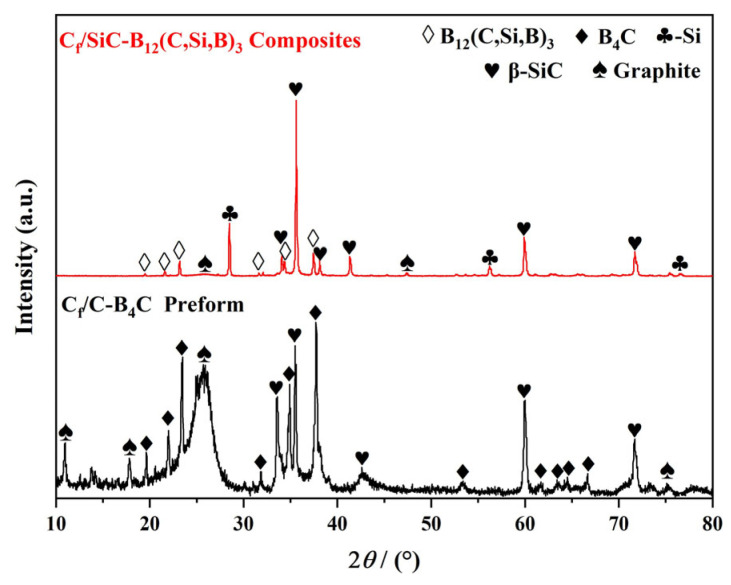
XRD patterns of the C*_f_*/C-B_4_C preform and C*_f_*/SiC-B_12_(C,Si,B)_3_ composite.

Figure 3 presents the XRD patterns of the intermediate C*_f_*/C-B_4_C preform (before RMI) and the final C*_f_*/SiC-B_12_(C,Si,B)_3_ composite (after RMI). The preform pattern shows diffraction peaks corresponding to B_4_C, β–SiC, and poorly crystallized graphite. The β–SiC signal originates from the SiC interphase deposited on the carbon fiber surface, while the broad graphite like diffraction peak is attributed to the carbon fibers, the PyC interphase, and the infiltrated carbon black. After RMI, the pattern of the composite reveals characteristic peaks of B_12_(C,Si,B)_3_, β–SiC, Si and graphite. Notably, the B_4_C phase in the preform is completely transformed into B_12_(C,Si,B)_3_ (PDF#19-0178), and β–SiC becomes the dominant crystalline phase, as evidenced by its significantly enhanced peak intensity.

These phase transformations result from the following reactions occurring during RMI [48]:

Si(l) + C(s) → SiC(s)(3)

15B_4_C(s) + 15Si(l) → 4B_12_(C,Si,B)_3_(s) + 3SiC(s) (4)

Quantitative analysis using the mixed-acid etching method revealed that the residual silicon content in the final composite is 19.3 vol%, confirming that silicon was introduced in excess during the reactive melt infiltration process. Importantly, no diffraction peaks corresponding to B_4_C are observed in the XRD pattern of the composite (Figure 3). These results collectively support the conclusion that almost all of the B_4_C was converted into B_12_(C,Si,B)_3_ and β–SiC phases during the RMI process, consistent with the reaction described in Equation (4).

During infiltration, molten silicon is driven by capillary forces into the porous preform. It reacts with carbon via Equation (3) a classic reaction-sintering process-to form β–SiC. Simultaneously, molten silicon reacts with B_4_C through Equation (4) to produce B_12_(C,Si,B)_3_ and additional β–SiC. The latter reaction follows a dissolution-reprecipitation mechanism: in the Si-saturated liquid, B_4_C particles dissolve, allowing Si atoms to enter the B_4_C lattice and replace C atoms in the C–B–C chains, thereby forming B_12_(C,Si,B)_3_ [38]. Excess molten silicon fills the remaining pores and solidifies as a residual Si phase upon cooling [49,50]. Furthermore, the consumption of carbon black by Equation (3) explains the marked decrease in the intensity of the graphite diffraction peaks in the composite.

### 3.2. Microstructural Observations

**Figure 4:**
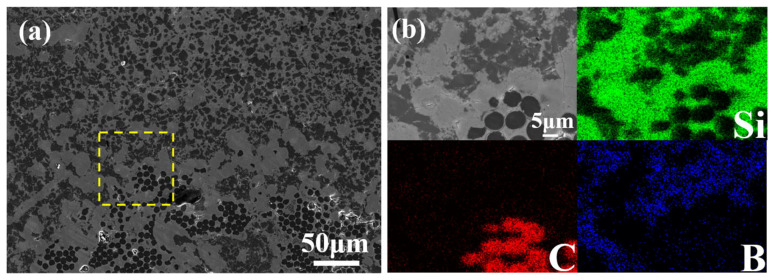
(**a**) SEM image of a polished cross-section of the C*_f_*/SiC-B_12_(C,Si,B)_3_ composite. (**b**) Magnified view of a selected region and the corresponding EDS elemental maps for Si, C, and B.

Figure 4a shows an SEM image of a polished cross-section of the composite. The contrast variation mainly arises from differences in atomic number among the phases: darker regions correspond to lower atomic-number elements, while lighter regions indicate higher atomic-number elements. The microstructure is highly dense and relatively uniform, with black polygonal and circular phases embedded in a gray continuous matrix.

A magnified view of a selected area and the corresponding EDS elemental maps for Si, C and B are presented in Figure 4b. The black circular features are carbon fibers, around which a distinct PyC/SiC hybrid interphase is clearly visible. This interphase remains well preserved and effectively protects the carbon fibers from erosion by molten silicon during RMI. The ceramic matrix region consists of black polygonal B_12_(C,Si,B)_3_ grains, dark-gray β–SiC phases, and light-gray residual silicon.

It should be noted that carbon is not homogeneously distributed in the composite microstructure. In the C*_f_*/SiC-B_12_(C,Si,B)_3_ composite, carbon originates from four primary sources: the carbon fibers, the pyrolytic carbon (PyC) interphase, the SiC interphase, and the SiC-B_12_(C,Si,B)_3_ ceramic matrix. Among these, the carbon fibers and PyC interphase consist solely of carbon, whereas the SiC interphase and the ceramic matrix (comprising β–SiC and B_12_(C,Si,B)_3_) also contain carbon, albeit in lower proportions. As shown in the EDS maps in Figure 4b, carbon is predominantly concentrated in the dark circular regions corresponding to the cross-sections of the carbon fibers. The 2.5D woven architecture of the carbon fiber preform inherently results in an inhomogeneous distribution of carbon fibers, with fiber bundles separated by matrix filled regions.

**Figure 5:**
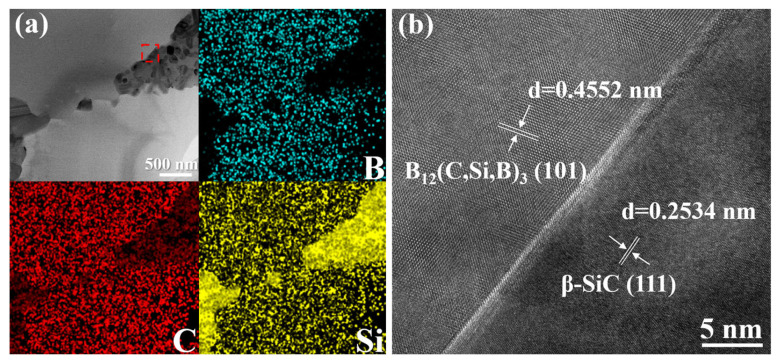
TEM analysis of the ceramic matrix of the C*_f_*/SiC-B_12_(C,Si,B)_3_ composite: (**a**) bright-field TEM image and corresponding EDS elemental maps of a representative ceramic matrix region; (**b**) high-resolution TEM image of the area marked by the red square in (**a**).

To clearly distinguish the B_12_(C,Si,B)_3_ and β–SiC within the ceramic matrix and to characterize their interfacial structure, transmission electron microscopy (TEM) coupled with energy-dispersive spectroscopy (EDS) was performed on the ceramic matrix region of the C*_f_*/SiC-B_12_(C,Si,B)_3_ composite. The corresponding bright-field TEM image and EDS elemental maps are presented in Figure 5a.

In the TEM image, two distinct phases are observed, exhibiting different contrasts due to variations in their average atomic numbers, appearing as brighter and darker regions. The EDS mapping confirms that the brighter regions are enriched in B and C, along with a certain amount of Si, and are thus reasonably identified as the B_12_(C,Si,B)_3_ phase. In contrast, the darker regions are enriched in Si and C but contain no B, suggesting they correspond to the β–SiC phase.

To further validate this assignment, high-resolution TEM (HRTEM) was conducted at the interface between the brighter and darker phases, as shown in Figure 5b. Both phases exhibit well-defined lattice fringes and a clean interface. The lattice spacing measured in the upper-left region is 0.4552 nm, which closely matches the (101) plane of B_12_(C,Si,B)_3_ (PDF#19-0178, standard d-spacing: 0.4550 nm). Meanwhile, the lattice spacing in the lower-right region is 0.2534 nm, corresponding well to the (111) plane of β–SiC (PDF#29-1129, standard d-spacing: 0.2520 nm). These observations confirm that both the B_12_(C,Si,B)_3_ and β–SiC phases formed via reactive melt infiltration are well crystallized.

### 3.3. Friction and Wear Behavior

**Figure 6:**
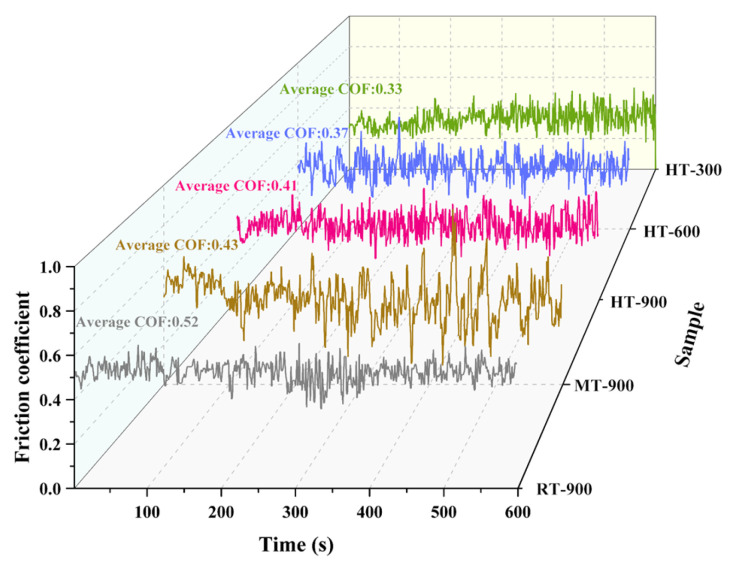
Friction coefficient curves of the C*_f_*/SiC-B_12_(C,Si,B)_3_ composite as a function of time under coupled temperature-velocity conditions. The corresponding average friction coefficients are also indicated.

Figure 6 presents the friction coefficient curves of the C*_f_*/SiC-B_12_(C,Si,B)_3_ composite as a function of time under coupled temperature-velocity conditions. When tested at a fixed rotational speed of 900 r/min, the average friction coefficient decreased from 0.52 at 25 °C, to 0.43 at 400 °C, and further to 0.41 at 600 °C. It is worth noting that the curve at 400 °C shows significant fluctuations, implying a possible transition of the friction mechanism at this temperature.

When the tests were conducted at a fixed temperature of 600 °C, the average friction coefficient increased slightly with rising rotational speed: 0.33 at 300 r/min, 0.37 at 600 r/min, and 0.41 at 900 r/min. All curves at 600 °C remained relatively stable over time. These results demonstrate that temperature exerts a stronger influence on friction behavior than rotational speed. The composite shows excellent friction stability at both 25 °C and 600 °C, whereas its performance degrades at 400 °C, likely due to the insufficient formation of surface lubricating phases. The underlying temperature driven evolution of surface composition and structure will be discussed in detail later.

**Figure 7:**
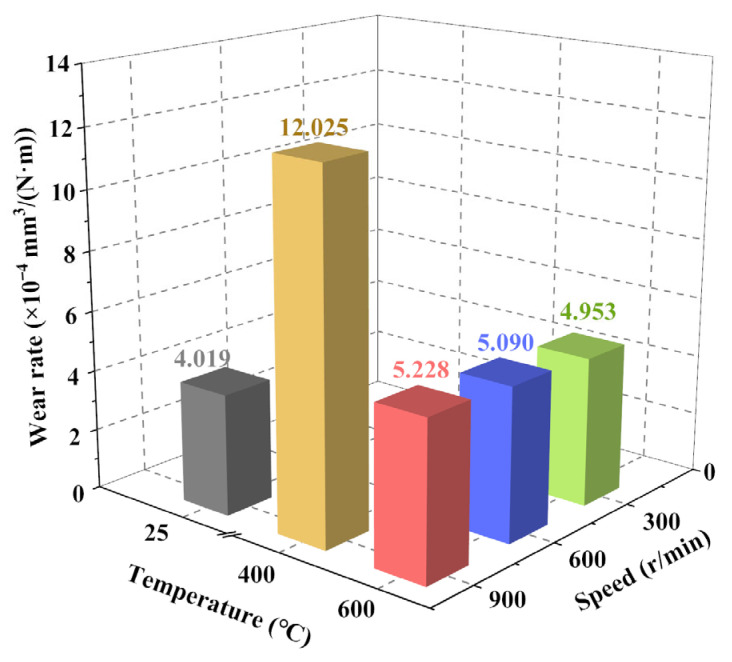
Volumetric wear rate of the C*_f_*/SiC-B_12_(C,Si,B)_3_ composite after ball-on-disk friction under variable temperature and velocity conditions. Note: The different colored bars in the figure represent different specimens: dark grey for RT-900, brownish-yellow for MT-900, red for HT-900, blue for HT-600, green for HT-300.

Figure 7 summarizes the volumetric wear rates of the composite after ball-on-disk tests, revealing a significant correlation between wear behavior and temperature. At room temperature, the composite exhibits the lowest wear rate (4.019 × 10^−4^ mm^3^/(N·m)). When the ambient temperature rises to 400 °C and 600 °C, the wear rates reach 12.025 × 10^−4^ mm^3^/(N·m) and 5.228 × 10^−4^ mm^3^/(N·m), increasing by approximately 199% and 30%, respectively, relative to the room-temperature value. The abnormally high wear rate at 400 °C, combined with the fluctuating friction coefficient, indicates a distinct tribological regime that severely degrades service performance.

Under a fixed high temperature of 600 °C, the wear rate shows minor variations with sliding speed: 4.953 × 10^−4^ mm^3^/(N·m) at 300 r/min, 5.090 × 10^−4^ mm^3^/(N·m) at 600 r/min, and 5.228 × 10^−4^ mm^3^/(N·m) at 900 r/min, increasing by approximately 2.8% and 5.6% relative to 300 r/min, respectively. This limited variation confirms that sliding speed exerts only a secondary influence compared with temperature.

Overall, temperature is the key factor governing the wear behavior of the C*_f_*/SiC-B_12_(C,Si,B)_3_ composite. The temperature of 400 °C represents a critical point where wear resistance is the poorest among the tested conditions.

The above results provide a comparative overview of the tribological performance of the C*_f_*/SiC-B_12_(C,Si,B)_3_ composite under different service conditions. At room temperature (25 °C, 900 r/min), the composite exhibits the highest friction coefficient (0.52) and the lowest wear rate (4.019 × 10^−4^ mm^3^/(N·m)). This combination is favorable for routine braking operations, such as light braking during normal driving, where stable friction and minimal material loss are essential to ensure braking safety under conventional conditions.

At elevated temperatures under a sliding speed of 900 r/min, the composite demonstrates a distinct performance profile. The friction coefficient at 600 °C (0.41) is lower than that at 400 °C (0.43), while the wear rate at 600 °C (5.228 × 10^−4^ mm^3^/(N·m)) is significantly lower than that at 400 °C (12.025 × 10^−4^ mm^3^/(N·m)). Notably, under the most severe condition (600 °C, 900 r/min), which simulates emergency braking scenarios where frictional heating rapidly elevates the material temperature, the composite maintains a high friction coefficient (0.41) and a relatively low wear rate. These characteristics are critical for ensuring braking safety during high-speed emergency stops, where both high friction and adequate wear resistance are required.

Taken together, the composite offers tailored tribological properties that meet the demands of both routine and emergency braking conditions, highlighting its potential as a high-performance braking material.

### 3.4. Surface Chemistry and Microstructural Evolution

**Figure 8:**
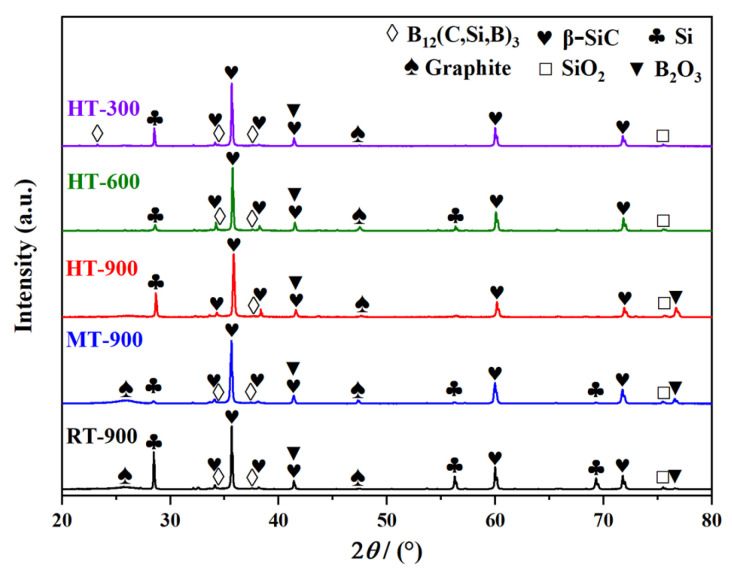
XRD spectra of the C*_f_*/SiC-B_12_(C,Si,B)_3_ composite after ball-on-disk friction under coupled temperature–velocity conditions.

Figure 8 summarizes the XRD patterns of the worn surfaces of the C*_f_*/SiC-B_12_(C,Si,B)_3_ composite after ball-on-disk friction tests under coupled temperature-velocity conditions. Compared to the as fabricated composite (Figure 3), the relative diffraction intensities of B_12_(C,Si,B)_3_ and Si are markedly reduced on all worn surfaces. At the same time, diffraction peaks corresponding to B_2_O_3_ and SiO_2_ appear under every test condition.

It is well established that during friction, intense shear and local high temperatures can readily promote the amorphization of crystalline phases at the surface. This phenomenon is particularly pronounced under the transient thermal shock induced by frictional heating and rapid cooling. In the present composite, phases such as B_12_(C,Si,B)_3_ and Si are susceptible to this effect. Simultaneously, sustained thermal accumulation drives oxidation reactions, leading to the formation of B_2_O_3_ and SiO_2_ [51,52]. These reactions likely occur via the following processes:

4B_12_(C,Si,B)_3_(s) + 63O_2_(g) → 30B_2_O_3_(s or l) + 12SiO_2_(s) + 12CO(g) (5)

2SiC(s) + 3O_2_(g) → 2SiO_2_(s) + 2CO(g) (6)

Si(s) + O_2_(g) → SiO_2_(s) (7)

Furthermore, compared with the as-fabricated sample, a broad diffraction “hump” of poorly crystallized graphite near 26° can still be observed on the worn surfaces of specimens tested at 25 °C and 400 °C (RT-900, MT-900). In contrast, this graphite-related diffraction feature becomes significantly weaker when the friction temperature reaches 600 °C, irrespective of rotational speed (HT-300, HT-600, and HT-900), although it remains detectable by XRD. This result indicates that the high-temperature environment significantly accelerates the oxidation of surface carbon, leading to a substantial reduction in its content, likely through the following reaction:

2C(s) + O_2_(g) → 2CO(g) (8)

C(s) + O_2_(g) → CO_2_(g) (9)

It should be emphasized that the formation of CO and CO_2_ does not improve the wear resistance of the composite. Instead, the oxidation of the carbon-rich lubricating phase to gaseous products leads to the loss of solid lubrication, which is detrimental to wear performance. It is worth noting that no diffraction peaks corresponding to H_3_BO_3_ were detected in any of the post-test XRD patterns, confirming that B_2_O_3_ remained stable on the composite surface without significant hydration under the current testing and sample handling conditions.

**Figure 9:**
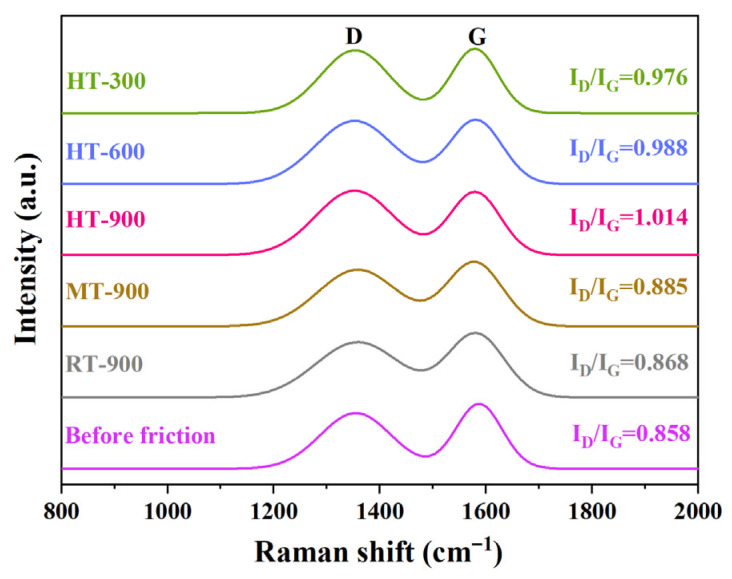
Raman spectroscopy analysis of the carbon phase on surface of C*_f_*/SiC-B_12_(C,Si,B)_3_ composites before and after friction. Note: In the figure, D and G represent the D peak and G peak of carbon, respectively.

To investigate the graphitization degree and structural evolution of carbon phases during friction, Raman spectroscopy was employed to characterize the carbon phases in C*_f_*/SiC-B_12_(C,Si,B)_3_ composites before and after friction, with the results presented in Figure 9.

All specimens exhibit distinct characteristic peaks at approximately 1350 cm^−1^ and 1580 cm^−1^, corresponding to the D band and G band, respectively [53]. It is well established that a higher *I_D_*/*I_G_* ratio indicates a lower degree of graphitization of the carbon phase [54,55]. The initial *I_D_*/*I_G_* ratio of the as-received composite is 0.858. After friction testing, this ratio slightly increases to 0.868 for Sample RT-900, 0.885 for Sample MT-900, 1.014 for Sample HT-900, 0.988 for Sample HT-600, and 0.976 for Sample HT-300, respectively.

Comparison of the *I_D_*/*I_G_* ratios reveals that both elevated temperature and increased sliding speed reduce the graphitization degree of the carbon phase on the composite surface. This phenomenon can be primarily attributed to two factors: on one hand, the carbon phase on the composite surface undergoes oxidation via reactions (8) and (9); on the other hand, the compressive and shear stresses induced by friction promote the disruption of the ordered graphite structure, leading to increased disorder. High temperature and high sliding speed further intensify these mechanisms, thereby exacerbating the reduction in graphitization degree of the carbon phase.

**Figure 10:**
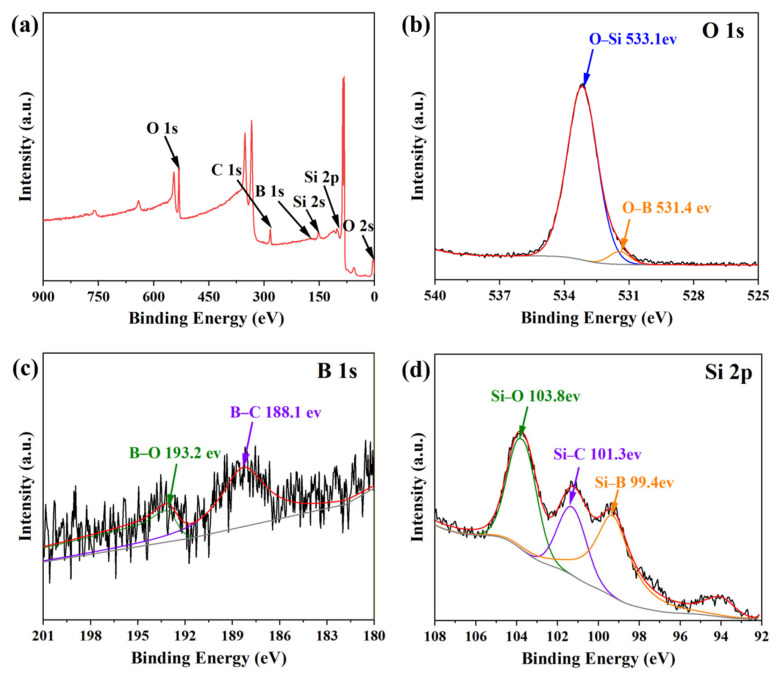
XPS spectra of the worn surface of Sample HT-900: (**a**) full survey scan; (**b**) O 1s high-resolution spectrum with peak-fitting; (**c**) B 1s high-resolution spectrum with peak-fitting; (**d**) Si 2p high-resolution spectrum with peak-fitting. Note: The red line in (**a**) represents the measured XPS survey spectrum. In (**b**–**d**), the black, red, and grey lines correspond to the raw experimental spectrum, the fitted spectrum, and the baseline, respectively.

The chemical bonding states on the worn surface of sample HT-900 were examined by XPS, as shown in Figure 10. The survey spectrum (Figure 10a) confirms that the worn surface consists of four elements: O, Si, C and B. The presence of oxygen clearly indicates surface oxidation of the C*_f_*/SiC-B_12_(C,Si,B)_3_ composite during the friction test [56,57].

The high-resolution O 1s spectrum (Figure 10b) exhibits two characteristic peaks. Peak-fitting analysis assigns binding energies of 533.1 eV and 531.4 eV to O–Si and O–B bonds, respectively.

High-resolution B 1s and Si 2p spectra (Figure 10c,d) exhibit, in addition to the oxygen-associated B–O (193.2 eV) and Si–O (103.8 eV) peaks, distinct signals corresponding to B–C (188.1 eV), Si–C (101.3 eV), and Si–B (99.4 eV) bonds. These features are attributed to the underlying B_12_(C,Si,B)_3_ and β–SiC phases that persist in the near-surface region [58,59,60,61].

These results demonstrate that under the combined influence of high temperature and shear stress, a tribochemical Si-B-O reaction layer, composed primarily of B_2_O_3_ and SiO_2_, is formed in situ on the worn surface. At the same time, the friction film is not fully oxidized; residual B_12_(C,Si,B)_3_ and β–SiC hard phases persist beneath the oxide layer. These retained hard phases help preserve the structural integrity of the friction interface, thereby supporting the wear resistance of the composite under severe testing conditions (up to 600 °C and 900 r/min).

**Figure 11:**
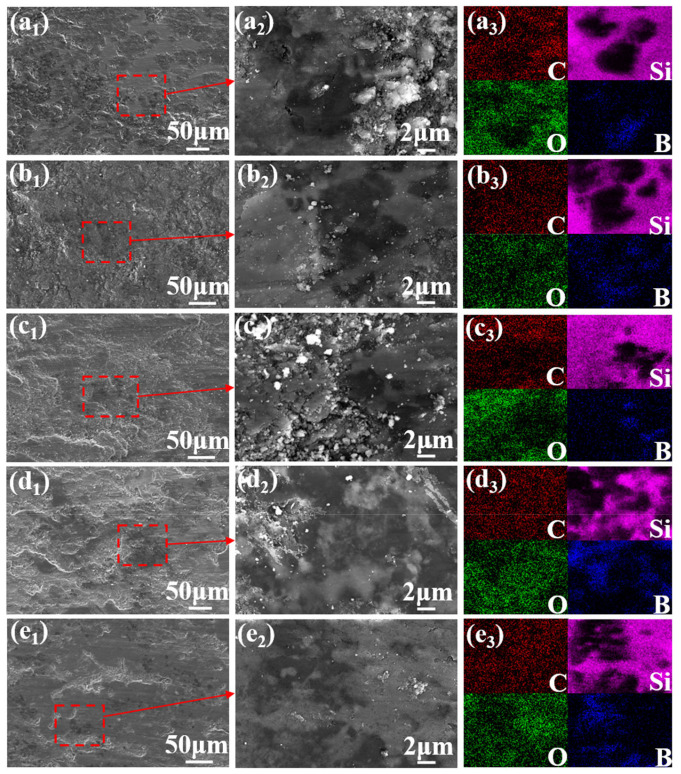
SEM images and EDS elemental maps (C, Si, O, B) of worn surfaces of the C*_f_*/SiC-B_12_(C,Si,B)_3_ composite after ball-on-disk friction. (**a_1_**) RT-900 specimen; (**a_2_**) magnified view of (**a_1_**); (**a_3_**) EDS maps of (**a_2_**); (**b_1_**) MT-900 specimen; (**b_2_**) magnified view of (**b_1_**); (**b_3_**) EDS maps of (**b_2_**); (**c_1_**) HT-900 specimen; (**c_2_**) magnified view of (**c_1_**); (**c_3_**) EDS maps of (**c_2_**); (**d_1_**) HT-600 specimen; (**d_2_**) magnified view of (**d_1_**); (**d_3_**) EDS maps of (**d_2_**); (**e_1_**) HT-300 specimen; (**e_2_**) magnified view of (**e_1_**); (**e_3_**) EDS maps of (**e_2_**).

Figure 11 presents SEM images of the worn surfaces of the C*_f_*/SiC-B_12_(C,Si,B)_3_ composite, along with the corresponding EDS elemental maps for C, Si, O, and B, respectively. At room temperature (RT-900, Figure 11(a_1_–a_3_)), the friction film is dense and flat. EDS mapping reveals slight local enrichments of carbon and oxygen, indicating that the film consists mainly of a carbon rich layer (likely originating from crushed carbon fibers) and a small amount of SiO_2_ formed by oxidation of residual silicon, while oxidation of β–SiC and B_12_(C,Si,B)_3_ is not pronounced.

When the friction temperature rises to 400 °C (MT-900, Figure 11(b_1_–b_3_)), randomly distributed pits appear, leading to an island-like, discontinuous friction film. This morphology originates from the partial oxidative consumption of surface carbon during reciprocating sliding. Meanwhile, the SiO_2_ and B_2_O_3_ formed by the oxidation of residual silicon and B_12_(C,Si,B)_3_ are insufficient in content and are mechanically removed under cyclic loading, thus failing to form a continuous and dense protective layer.

At 600 °C (HT-900, Figure 11(c_1_–c_3_)), oxygen becomes significantly enriched, confirming intensified surface oxidation. At this temperature, the surface carbon is significantly consumed, and the friction film transforms into a composite oxide of SiO_2_ and B_2_O_3_ produced by the high-temperature oxidation of Si, β–SiC, and B_12_(C,Si,B)_3_. This oxidation can form a continuous and stable Si-B-O high-temperature lubricating film.

Comparing samples HT-900, HT-600, and HT-300 (Figure 11(c_1_–e_1_)) further shows that as the rotational speed increases, localized peeling of the friction film becomes more apparent. This is attributed to higher shear stress at elevated speeds. Such shear stress promotes fatigue delamination of the surface film and the underlying hard phases (β–SiC and B_12_(C,Si,B)_3_) under cyclic loading. As a result, surface damage accumulation is accelerated [22].

**Figure 12:**
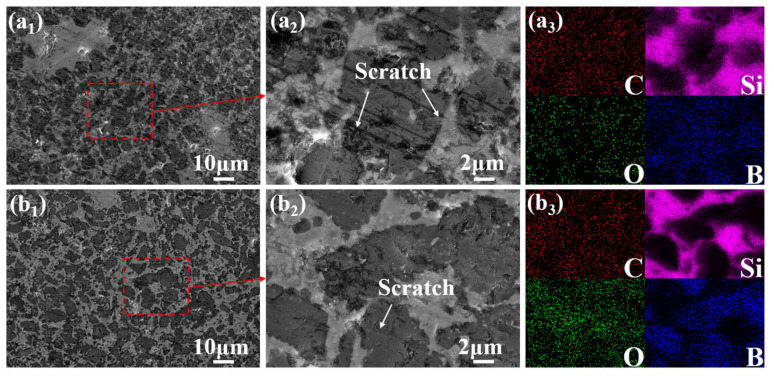
Surface morphologies and EDS elemental maps of the C*_f_*/SiC-B_12_(C,Si,B)_3_ composite before and after static oxidation and friction tests. (**a_1_**–**a_3_**) As-polished surface; (**b_1_**–**b_3_**) after static oxidation at 600 °C for 20 min. Note: The red dashed boxes and arrows in the figure indicate the magnified microstructures of the selected regions.

To distinguish the contributions of thermal oxidation and friction-induced oxidation to the formation of the Si-B-O tribofilm, a static oxidation experiment was conducted on the composite at 600 °C for 20 min, and the surface morphology was compared with those of the as-polished (Figure 12), RT-900, and HT-900 specimens. The as-polished surface exhibits numerous mechanical scratches from sandpaper grinding, with no detectable oxygen enrichment (Figure 12(a_1_–a_3_)).

After static oxidation at 600 °C, the scratches are significantly weakened, and pronounced oxygen aggregation, primarily enriched in the scratched areas, is observed (Figure 12(b_1_–b_3_)). This indicates the formation of a Si-B-O oxide film. Notably, B_2_O_3_ (melting point ≈ 450 °C) exhibits fluidity at 600 °C, which contributes to the healing of surface scratches. In contrast, the HT-900 specimen (sliding at 600 °C) shows a fractured and accumulated oxide film morphology (Figure 11(c_1_–c_3_)), suggesting that mechanical disruption during sliding dominates over the healing effect of thermal oxidation under this condition. For the RT-900 specimen (sliding at room temperature), a relatively continuous friction film was observed, accompanied by slight oxygen enrichment (Figure 12(a_1_–a_3_)). This oxide formation is attributed to transient flash temperatures generated during friction rather than ambient thermal oxidation. These observations collectively indicate that at 600 °C, ambient thermal oxidation plays a dominant role in oxide film formation, while the contribution of friction-induced oxidation is comparatively limited under the present testing conditions.

**Figure 13:**
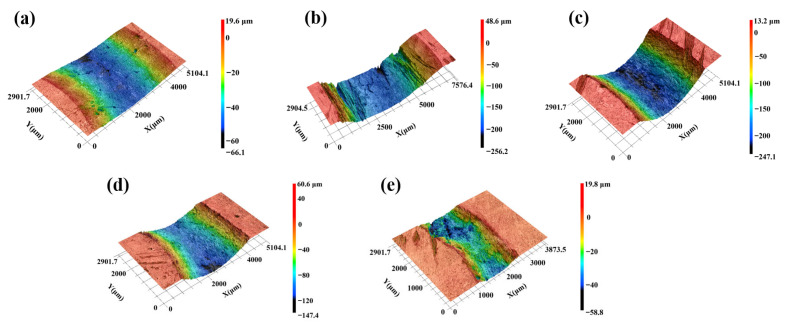
Three-dimensional profiles of worn regions of C*_f_*/SiC-B_12_(C,Si,B)_3_ composite after ball-on-disk friction: (**a**) RT-900; (**b**) MT-900; (**c**) HT-900; (**d**) HT-600; (**e**) HT-300. Note: The Z-axis scales are not uniform across subfigures due to the measurement constraints of the laser confocal microscope (VK-X1000, Keyence), which does not allow manual customization of the scale range. The color mapping in each subfigure corresponds directly to the measured depth profile of the respective specimen.

The three-dimensional profiles of the worn regions on the C*_f_*/SiC-B_12_(C,Si,B)_3_ composite after ball-on-disk friction tests were obtained using laser confocal microscopy, as shown in Figure 13. These profiles provide complementary depth-resolved morphological information to the surface SEM observations shown in Figure 11. At room temperature (RT-900, Figure 13a), the wear track is relatively smooth and exhibits distinct abrasive grooves, with a maximum wear depth of 66.1 μm. During sliding, surface asperities are sheared and compacted into a continuous, dense carbon-rich film-as previously noted, primarily originating from crushed carbon fibers-which effectively shields the underlying matrix. This protective layer accounts for the friction coefficient curve with a narrow fluctuation range (Figure 6) and the low wear rate (4.019 × 10^−4^ mm^3^/(N·m)) measured previously for sample RT-900.

When the friction temperature rises to 400 °C (MT-900, Figure 13b), the surface exhibits pronounced pits and protrusions, and the wear depth increases sharply to 256.2 μm. This morphology aligns with the discontinuous island-like friction film observed in Figure 11(b_1_–b_3_), where partial oxidation of carbon and insufficient formation of SiO_2_ and B_2_O_3_ lead to inadequate surface protection. Consequently, larger fluctuations in friction coefficient (average 0.43) and a significantly higher wear rate (12.025 × 10^−4^ mm^3^/(N·m)) are observed.

Under high-temperature conditions at 600 °C (HT-900, Figure 13c), the wear depth reaches 247.1 μm, and the morphology transitions to a discontinuous lamellar oxide film accompanied by accumulated abrasive particles. Micro-grooves formed by the plowing effect of these particles can be observed in the film areas, consistent with the features shown in Figure 11(c_1_,c_2_). Furthermore, high temperature and high sliding speed synergistically accelerate the tribo-oxidation reaction on the surface: Si and β–SiC oxidize to SiO_2_, while B_12_(C,Si,B)_3_ oxidizes to B_2_O_3_ and SiO_2_. These oxides have low mechanical strength; some spall off during sliding, while the remainder is crushed and acts as a solid lubricant, effectively reducing the interfacial mechanical resistance [56,62]. Therefore, the average friction coefficient of HT-900 (0.41) is lower than those of RT-900 (0.52) and MT-900 (0.43).

As the rotational speed decreases at 600 °C (HT-600 and HT-300, Figure 13d,e), the maximum wear depth is reduced to 141.4 μm and 58.8 μm, respectively. The agglomeration of abrasive particles diminishes, and the coverage of the lamellar friction film increases (see also Figure 11(d_1_,e_1_)). Lower speeds reduce frictional heat accumulation, moderately suppress surface oxidation, and lessen mechanical scratching by abrasive particles, thereby lowering the tendency for film spallation. This leads to a smoother worn surface and consequent reductions in both friction coefficient (0.37 for HT-600, 0.33 for HT-300) and wear rate (5.090 × 10^−4^ mm^3^/(N·m) for HT-600, 4.953 × 10^−4^ mm^3^/(N·m) for HT-300).

**Figure 14:**
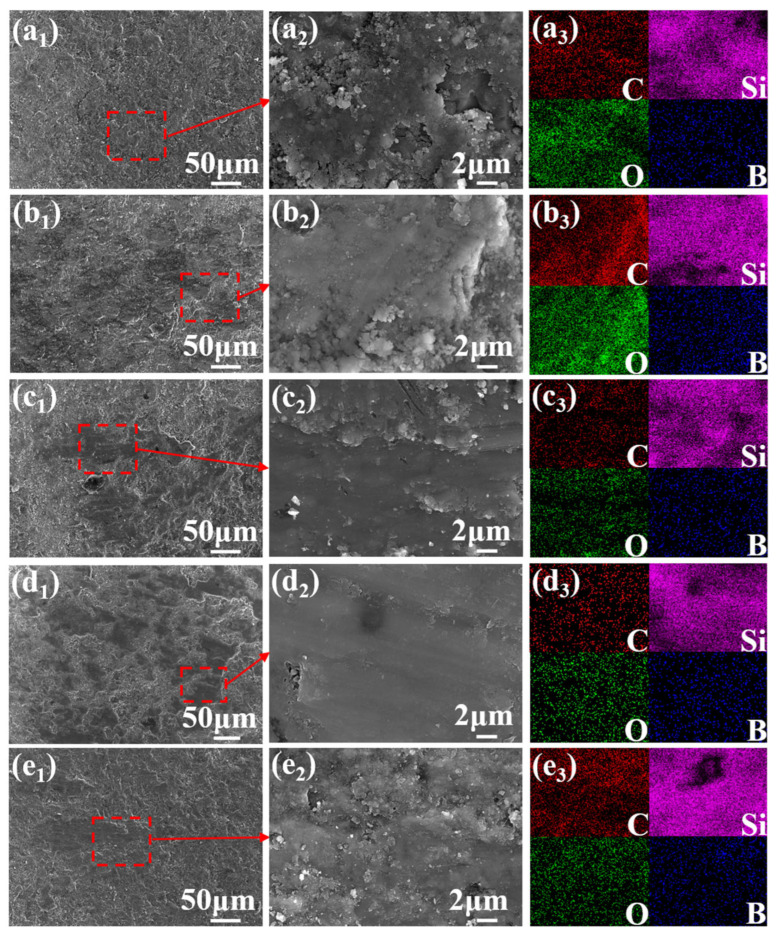
SEM micrographs and EDS elemental maps of the worn SiC counterbody surfaces after friction tests: (**a_1_**–**a_3_**) against RT-900; (**b_1_**–**b_3_**) against MT-900; (**c_1_**–**c_3_**) against HT-900; (**d_1_**–**d_3_**) against HT-600; (**e_1_**–**e_3_**) against HT-300. Note: The red dashed boxes and arrows in the figure indicate the magnified microstructures of the selected regions.

To further elucidate the tribological behavior of the C*_f_*/SiC-B_12_(C,Si,B)_3_ composite against the SiC counterpart, the worn surfaces of the SiC balls after friction tests under different conditions were examined by SEM and EDS. The results are presented in Figure 14.

At room temperature (Figure 14(a_1_–a_3_)), a flake-like, discontinuous tribofilm formed on the SiC ball surface, with a limited number of pits. Pronounced tearing marks were observed at the edges of the tribofilm, accompanied by local enrichment of oxygen and carbon. This indicates the transfer and subsequent oxidation of wear debris from the composite to the counterbody surface during sliding.

At the test temperature of 400 °C (900 r/min), the tribofilm on the SiC ball surface exhibited pronounced tearing and large-area detachment (Figure 14(b_1_,b_2_)). EDS elemental mapping revealed significant enrichment of oxygen and carbon at the edges of the detached regions, confirming the occurrence of local oxidation at the composite/counterbody interface and the transfer of the carbon-rich film from the composite to the SiC ball (Figure 14(b_3_)). Owing to the insufficient formation of lubricious oxides (B_2_O_3_ and SiO_2_) at this temperature, a continuous and stable tribofilm could not be established. As a result, hard phases on the SiC ball surface spalled off, generating hard third-body abrasive particles. These particles intensified the mechanical cutting and plowing action between the friction interfaces, leading to severe fluctuations in the friction coefficient (Figure 6) and the highest wear rate (12.025 × 10^−4^ mm^3^/(N·m)) among all tested conditions. This mechanism explains why the wear rate at 400 °C is approximately three times higher than that at room temperature.

After sliding against the HT-900 specimen at 600 °C (Figure 14(c_1_–c_3_)), a continuous and uniform oxide tribofilm formed on the SiC ball surface. The corresponding EDS elemental mapping showed a homogeneous distribution of oxygen within the tribofilm, indicating the establishment of a stable oxide layer.

As the rotational speed decreased to 600 r/min (Figure 14(d_1_–d_3_)), the tribofilm on the SiC ball transformed into an island-like, discontinuous distribution. Upon further reduction to 300 r/min (Figure 14(e_1_–e_3_)), the integrity of the tribofilm further deteriorated, and numerous powdery particles became embedded within it.

Notably, the integrity of the tribofilm on the SiC ball at 600 °C exhibited a trend opposite to that observed on the composite surface. This discrepancy arises because the tribofilm on the SiC ball consists primarily of SiC and its oxidation product, SiO_2_. Both materials have melting points exceeding 1600 °C and remain in the solid state at 600 °C. At low sliding speeds, the shear stress generated between the friction pair is relatively low, making it difficult to effectively comminute the SiC and SiO_2_ particles. As the sliding speed increases, the shear stress intensifies, producing a greater amount of wear debris, which is subsequently compacted into surface pits under cyclic loading, forming a more continuous tribofilm.

Interestingly, the friction coefficient of the friction pair at 600 °C increased slightly with rotational speed. Considering the evolution of the tribofilm morphology on the composite surface with varying speeds, it can be inferred that the C*_f_*/SiC-B_12_(C,Si,B)_3_ composite plays a dominant role in regulating the friction coefficient at this temperature. Furthermore, no agglomeration of boron was detected on any of the SiC ball surfaces, indicating that the transfer of B_2_O_3_ to the counterbody is not prominent during sliding.

### 3.5. Friction and Wear Mechanisms

**Figure 15:**
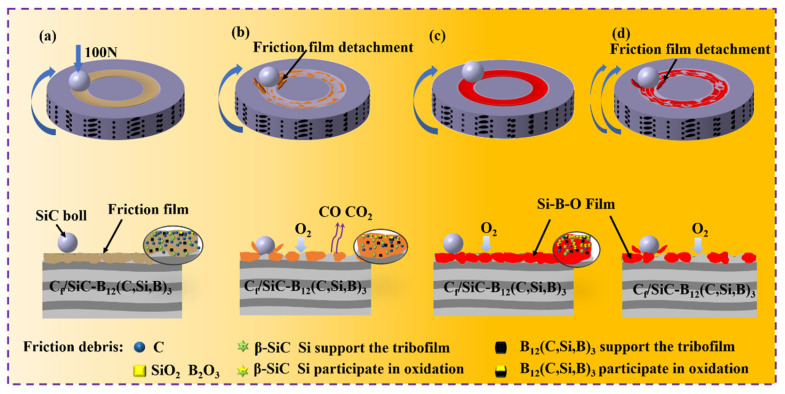
Schematic diagrams illustrating the friction and wear mechanisms of C*_f_*/SiC-B_12_(C,Si,B)_3_ composite under different temperature–velocity coupled conditions during ball-on-disk friction: (**a**) RT-900; (**b**) MT-900; (**c**) HT-300; (**d**) HT-900. Note: The blue arrows in the figure represent the rotation direction of the specimen during the test.

The friction and wear mechanisms of the C*_f_*/SiC-B_12_(C,Si,B)_3_ composite under different temperature-velocity conditions are schematically summarized in Figure 15. At room temperature (Figure 15a), surface asperities on both the composite and the SiC grinding ball are progressively sheared and pulverized under normal and tangential loading, generating substantial tribo-debris [62]. A portion of this debris is expelled, while the remainder fills surface micro-valleys and is compacted to form a relatively continuous friction film. During this process, graphitized carbon fibers are pulled out and crushed, producing a carbon-rich solid lubricating film between the sliding pairs. This film enhances coefficient-of-friction stability and mitigates delamination, resulting in the low friction and wear rates presented earlier.

At 400 °C (Figure 15b), the surface carbon film undergoes oxidation, generating gaseous CO and CO_2_ that escape from the interface. This process significantly degrades the self-lubricating capability of the composite. Concurrently, the oxide products formed from residual silicon and the B_12_(C,Si,B)_3_ phase namely SiO_2_ and B_2_O_3_ remain thermodynamically stable at this temperature, with negligible volatilization. However, the melting point of B_2_O_3_ (≈450 °C) is close to the ambient temperature, and frictional heating induces transient local temperatures above 400 °C. Under cyclic shear stress, B_2_O_3_ undergoes mechanical softening, leading to its rapid crushing and detachment. As a result, the dynamic formation rate of the oxide layer is lower than its mechanical removal rate, preventing the development of a continuous and dense protective oxide barrier [63,64]. Consequently, both the carbon film and the underlying hard phases experience localized detachment under cyclic loading, generating numerous surface pits. This leads to the largest fluctuations in friction coefficient (Figure 6) and the highest wear rate (Figure 7) among all tested conditions. The interpretation of 400 °C as a “critical point” is thus supported by the combined effects of carbon fiber oxidation (initiated at this temperature according to Tong et al. [65]), frictional heating, and the mechanically unstable nature of B_2_O_3_ near its melting point under shear.

It should be noted that the hard phases B_12_(C,Si,B)_3_ and β–SiC play a dual role during the friction process. A portion of these phases participates in the tribochemical reaction, oxidizing to form the Si-B-O oxide layer that provides lubrication and oxidation protection. The remainder remains intact beneath the reaction layer and serves as a load-bearing framework, mechanically supporting the tribofilm and preventing its premature collapse under cyclic shear stress. This dual function is schematically illustrated in Figure 15, where solid-color particles denote the load-bearing components, and dual-color particles denote those involved in the oxidation reaction.

A moderate temperature rise facilitates the formation of an oxide layer with solid lubricating properties [66]. At 600 °C under low sliding speed (Figure 15c), the coupled thermo-mechanical effect induces oxidation of Si, β–SiC, and B_12_(C,Si,B)_3_ phases, generating SiO_2_ and B_2_O_3_. Notably, B_2_O_3_ has a melting point of 450 °C and exhibits certain fluidity at this temperature [22]. Promoted by cyclic loading, it spreads together with SiO_2_ across the contact interface to form a continuous Si-B-O composite film. This film acts as both a solid lubricant and an oxygen diffusion barrier, improving lubricity and retarding further oxidation inside the composite. The reduction in friction coefficient at 600 °C is attributed to the combined effect of reduced shear strength and the diffusion barrier function of the Si-B-O oxide layer. The lower shear strength of SiO_2_ and B_2_O_3_ compared to the original hard phases directly decreases frictional resistance. Concurrently, the continuous oxide layer physically isolates surface asperities and suppresses oxygen diffusion into the composite interior, thereby reducing oxidative wear. The decrease in wear rate, however, is primarily governed by the diffusion barrier effect, as this prevents further oxidation-induced damage to the subsurface material.

With increasing rotational speed at 600 °C (Figure 15d), intensified mechanical abrasion and enhanced surface oxidation weaken the bonding strength of the Si-B-O film. Under cyclic loading, fatigue-induced spallation occurs, resulting in a fragmented and partially accumulated morphology as observed in Figure 11(c_1_). Oxygen penetrates into the composite through the spalled regions. Under the coupled effects of oxidation and mechanical action, the oxide film undergoes continuous formation and removal, establishing a sustained cycle. This ultimately leads to an increase in the wear rate, while the friction coefficient remains at a relatively low level.

## 4. Conclusions

In this work, a novel C*_f_*/SiC-B_12_(C,Si,B)_3_ composite was successfully synthesized via a hybrid SI-RMI process, and its tribological properties were preliminarily investigated under variable temperature (25, 400, 600 °C) and sliding speed (300, 600, 900 r/min) conditions. The key findings are as follows:

1. Temperature appears to be the dominant factor influencing friction and wear behavior. At room temperature, a carbon-rich lubricating film forms from crushed fibers, yielding a friction coefficient of 0.52 and a wear rate of 4.019 × 10^−4^ mm^3^/(N·m). At 400 °C, intensified carbon oxidation and insufficient oxide formation lead to a discontinuous surface film, associated with increased wear (12.025 × 10^−4^ mm^3^/(N·m)) and friction fluctuations. At 600 °C, a continuous in situ Si-B-O composite oxide layer (SiO_2_ and B_2_O_3_) forms, which may act as both a solid lubricant and an oxygen barrier, contributing to a lower friction coefficient (0.41) and reduced wear.
2. Under high-temperature conditions (600 °C), sliding speed appears to have a secondary influence. Increasing the rotational speed from 300 to 900 r/min increases the wear rate only slightly (from 4.953 × 10^−4^ to 5.228 × 10^−4^ mm^3^/(N·m)), suggesting that the high-temperature oxide film possesses some shear stability.
3. The friction film undergoes dynamic evolution with service conditions. At 600 °C, the film is predominantly composed of SiO_2_ and B_2_O_3_ originating from the oxidation of β–SiC, residual silicon, and the B_12_(C,Si,B)_3_ phase. This composite oxide layer may function not only as a friction-reducing lubricant but also as a barrier against further oxidation.
4. The in situ formed B_12_(C,Si,B)_3_ phase appears to play a role in high-temperature lubrication through its contribution to B_2_O_3_ formation. The transition from carbon-based to oxide-based lubrication likely underlies the composite’s ability to maintain performance under thermal-mechanical coupling.

This study provides a preliminary understanding of the tribological mechanisms of the C*_f_*/SiC-B_12_(C,Si,B)_3_ composite. Due to the single-measurement nature of the tests, the findings should be considered exploratory, and further statistical validation is recommended in future work.

## Data Availability

The data presented in this study are available on request from the corresponding author due to privacy and legal reasons.
